# Development and testing of the hemodialysis symptom distress scale (HSD-22) to identify the symptom cluster by using exploratory factor analysis

**DOI:** 10.1186/s12882-021-02337-7

**Published:** 2021-04-12

**Authors:** Mei-Chu Chen, Ya-Fang Ho, Chiu-Chu Lin, Chia-Chen Wu

**Affiliations:** 1 Department of Nursing, Kaohsiung Chang Gung Memorial Hospital, Kaohsiung, Taiwan; 2 School of Nursing, Kaohsiung Medical University, Kaohsiung, Taiwan; 3 School of Nursing, China Medical University, Taichung, Taiwan; 4 Department of Medical Research, Kaohsiung Medical University Hospital, Kaohsiung, Taiwan; 5 Department of Renal Care, College of Medicine, Kaohsiung Medical University, No. 100, Shih-Chuan 1st Road, Kaohsiung, 807 Taiwan; 6 School of Nursing, Fooyin University, Kaohsiung, Taiwan

**Keywords:** Exploratory factor analysis, Hemodialysis, Scale, Symptom distress, Symptom cluster

## Abstract

**Background:**

Patients receiving hemodialysis (HD) often experience multiple symptoms concurrently and these symptoms may impact their quality of life. A valid and reliable tool is needed to assess the symptom distress of patients receiving HD in terms of the perspective of symptom clusters. Although many studies have explored symptom clusters related to patients receiving HD, the clusters formed had problems with overlapping, vagueness, lack of cluster-specificity, and difficulty in discerning their common mechanism under the cluster.

**Aims:**

To develop reliable measurement tool to identify the symptom clusters of patients undergoing HD.

**Design:**

A cross-sectional descriptive study.

**Methods:**

To examine the physiological properties of the HD symptom distress (HSD) scale, 216 participants were recruited from a HD center of a medical university hospital in southern Taiwan from February 2019 to April 2019. Construct validity was evaluated by exploratory factor analysis (EFA), and the internal consistency and test–retest reliability were estimated by Cronbach’s alpha and intraclass correlation coefficient (ICC).

**Results:**

The CVI value of the HSD was 0.89. The HSD scale was composed of five factors with 22 items, including insufficient energy/vitality, cardiac–pulmonary distress, sleep disturbance, musculoskeletal distress, and gastrointestinal distress, with factor loading ranging from 0.62 to 0.87, explaining 65.5% of the total variance. Cronbach’s alpha coefficient of the HSD total scale was 0.93, and five subscales ranged from 0.73 to 0.89. The test-retest reliability was 0.92 (*p* < 0.001) by using the intraclass correlation coefficient (ICC) for the HSD-22 scale.

**Conclusion / implication:**

Theoretical testing from our study indicated that the HSD-22 scale had satisfactory validity and reliability. Therefore, this assessment tool can be employed to identify the symptom clusters of patients receiving HD in the clinical setting. Such identification enables healthcare professionals to provide interventions to release patients’ symptom distress efficiently.

**Supplementary Information:**

The online version contains supplementary material available at 10.1186/s12882-021-02337-7.

## Background

In accordance with the United States Renal Data System (USRDS) Report, the global population with end-stage kidney disease (ESKD) has increased by nearly 20% since 2000; and among them, the prevalence of received renal replacement therapy with hemodialysis (HD) in the US increased more than 80% from 2000 to 2017 [1]. The incidence of ESKD is rising in Taiwan. The prevalence of dialysis had increased from 1448 per million of the population in 2000 to 3480 in 2017, 90% of them with HD. There were 3251cases per million of the population undergoing dialysis, ranking the highest in the world in 2016 [2]. HD was the primary treatment for patients with ESKD in Taiwan. However, a considerable number of patients still suffered from multiple symptoms of distress due to HD treatment [3–5]. HD may cause to poor quality of life, as patients with ESKD on dialysis suffered from a severe symptom burden caused by the disease itself, the treatment, and comorbid conditions [3, 6].. The multiple symptoms experienced by patients receiving HD were reported including tiredness, sleep disturbance, dry mouth, muscle weakness, pruritus [4, 5, 7], insomnia, nausea, anorexia, and shortness of breath [7, 8]. Furthermore, Fidan et al. reported that almost all patients receiving HD had one or more musculoskeletal problems, the most common of which were muscle cramps, myalgias and arthralgias [9]. The study of Flythe et al. (2018) demonstrated the most common physical distresses and symptoms in patients receiving HD were fatigue, cramping, and body aches [10]. In addition to the physical symptoms, depression, anxiety, feeling worried, and frustration had commonly occurred in patients on HD [9–13]. However, there is increased evidence to support the idea that symptoms of patients with ESKD occurred in groups concurrently; so-called symptom clusters. Although studies have explored symptom clusters related to patients receiving HD [7, 12, 14], the clusters formed had problems with overlapping, vagueness, and lack of cluster-specificity, and it was difficult to identify their common mechanism under the cluster. The report of participants from 27 states in the United States indicated that frequency and duration of symptoms, as well as unpredictability effects factored most heavily into symptom prioritization [10]. This study applied the Theory of Unpleasant Symptoms (TOUS) theoretical foundation to describe the coexistence and interaction of multiple symptoms. Symptoms can occur in isolation, but two or more symptoms can occur simultaneously. A Taiwanese study about symptom clusters among HD patients presented in 2012 reported that it is important to explore different regions for HD patients and symptom management. The reason is that northern HD patients might present different symptoms from southern patients due to their different life styles. Previous studies explored the prevalence and severity of symptoms. However, scant studies examined the daily living distress derived from these symptoms in clinical settings. A valid and reliable tool is needed to assess the symptom distress of hemodialysis patients in terms of the perspective of symptom clusters.

## Method

### Study design, settings and participants

**Fig. 1:**
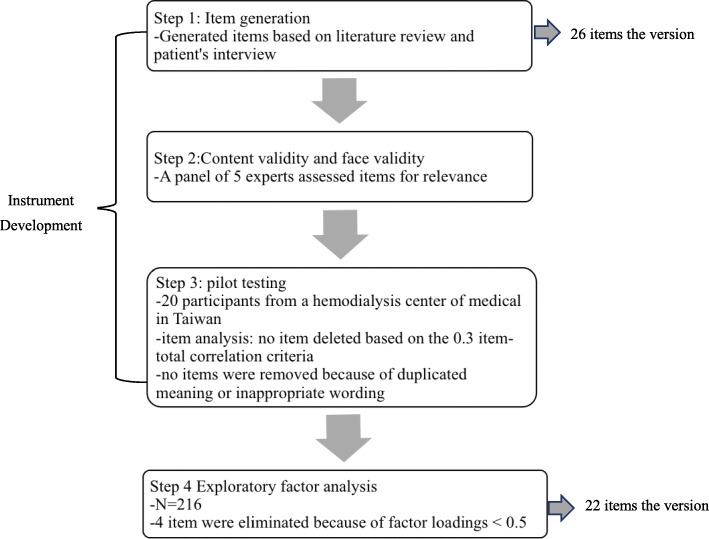
Process of developing and validating the HSD

Hemodialysis Symptom Distress (HSD) instrument was established steps (Fig. 1). Steps I: Item generation. Steps II: Content validity and face validity. Steps III: Pilot study. To verify the validity of the construct, exploratory factor analysis (EFA) was performed to determine factor structure. We conducted a cross-sectional descriptive study. 216 participants were recruited from a HD center of a medical university hospital in southern Taiwan from February 2019 to April 2019. The recruitment criteria were participant over 20 years old who had been undergoing HD for ≥3 months, and able to comprehend and communicate in Mandarin Chinese or Taiwanese. Per the request of the study investigators, HD nursing staff referred participants from HD outpatient clinics to investigators for recruitment and two-hundred and sixteen participants participated in this study.

### Instrument development

#### Item generation

In order to generate an item pool, we had referred to previous literature [7, 12, 14–17]. Based on the existing literature and the experiences from patients receiving HD, 26 candidate items were generated and this formed an initial draft of the hemodialysis symptom distress (HSD) scale.

#### Determination of content validity

After the pool of candidate items had been developed, its content validity was tested by five experts including one nephrologist, two nurse educators, and two nurse practitioners working in a HD center of a medical university hospital, three of them with a PhD and specialized in instrument development and nephrology. These experts used a four-point Likert scale to rate the relevance and wording of each item, the scores were as follows: 1-least relevant, 2-somewhat relevant, 3-quite relevant and 4-most relevant. If an expert rated any item < 4, the expert was asked to provide his/ her suggestion for the item modification.

We used the content validity index (CVI) suggested to quantify the extent of expert scores and when the significant proportion of experts rated items as 3 or 4 points, it is retained [18]. The relevance of symptom distress and accuracy of each item in this initial draft were assessed by five experts. Four items with problematic wording were revised based on the recommendations of the panel to result in a final draft containing 26 items (i.e., the HSD-26). The CVI value of the HSD was 0.89.

#### Determination of face validity

To evaluate the face validity of the HSD scale, investigators administered the draft of the instrument (i.e., the HSD-26) to a convenient sample from the data collection sites in this study. Twenty patients who were receiving HD were invited to a pilot study of HSD-26 for clarity, comprehension and ease of response. The investigator selected patients who were similar to the research participants to test formally and actually answer in the future. Items were scored on a 4-point scale from 1 (never) to 4 (always). Total scores ranged from 26 to 104. Higher scores indicating severe symptom distress.

### Ethical considerations

The study was approved by the medical center Institutional Review Board. The clinical nurses in the dialysis center were asked to consult patients who met the inclusion criteria and the Principal Investigator explained the purpose and procedures of the study to the subject. Informed consent to participate in the study was obtained. After being recruited, participants needed to fill out the questionnaires for about 20 to 30 min, and confidentiality of collected data would be maintained. Participants were also assured that they could withdraw freely from the study at any time and for any reason.

### Data collection

They filled out the questionnaires during a HD session after receiving the written informed consent from participants. Data was collected from February to April in 2019. Consent forms were distributed to 220 patients and 216 patients signed their consent to participate this study (attrition rate = 1.9%).

### Data analysis

EFA was used to identify the factor structure. The Kaiser–Meyer–Olkin (KMO) test for sampling adequacy and Bartlett’s test for sphericity were performed and the number of factors to be retained was determined by parallel analysis [19]. Items selected met the following four criteria: (a) factor loading > 0.5; (b) minimum factor membership of three items; (c) no cross factor loaded items; and (d) conceptual coherence of items with its corresponding factor. Internal consistency was assessed by determining Cronbach’s alpha coefficients for the overall scale and subscales. A Cronbach’s alpha coefficient > 0.70 was considered satisfactory [20, 21].

## Results

Two-hundred and sixteen participants completed the HSD questionnaire, among them, 44.4% were male and 55.6% were female, with an age range of 20 to 88 years (mean = 63.0, SD = 12.75). The educational level of the participants was diverse (53.7% with elementary school or less; 34.3% with a high school diploma; 12.0% with a college degree), and the majority of participants (87.2%) were married (Table 1).

**Table 1:** Baseline characteristics (*N* = 216)

Variables	n (%)
Age (mean ± SD)	63 ± 12.75
Sex	
Male	96 (44.4%)
Female	120 (55.6%)
Marital status	
Married	324 (86.2%)
Single	47 (12.5%)
Widowed	2 (0.5%)
Divorced	1 (0.3%)
Unknown	2 (0.5%)
Education	
Elementary school or less	116 (53.7%)
High school diploma	74 (34.3%)
College degree	26 (12.0%)

*SD* standard deviation

### Exploratory factor analysis (EFA)

The factor structure of initial HSD was analyzed with a sample of 216 participants by using EFA. Factors were extracted by using principal component analysis, the correlation matrix and pairwise deletion method. The KMO measurement of sampling adequacy was 0.90 which indicated excellent sampling adequacy and relatively compact patterns of correlation. Such factor analysis should produce distinct and reliable factors [21]. Bartlett’s test of sphericity was significant (chi-square = 2588.812, *df* = 231, *p* < 0.000) which showed that it had an adequate relationship between the variables [22]. Oblique Promax rotation procedures were used as the method of factor rotation, because HSD scale factors were assumed to be correlated. Four items (items 12, 21, 23, and 24) were eliminated from the draft 26-item HSD due to a factor loading < 0.5. A five-factor solution for the 22 remaining items provided the most meaningful factor pattern and were labeled as: insufficient energy/vitality, cardiac–pulmonary distress, sleep disturbances, musculoskeletal distress, and gastrointestinal distress, with loading ranging from 0.62 to 0.87, explaining 65.5% of the total variance. Cronbach’s alpha coefficient of the HSD total scale was 0.93, and five subscales ranged from 0.73 to 0.89. The loading ranges of five factors are shown in Table 2 and the factor structures were described as following:

**Table 2:** Exploratory factor analysis (EFA) results and Cronbach’s alpha coefficients

Symptom	Factor loading					Cronbach’s alpha
	Factor 1	Factor 2	Factor 3	Factor 4	Factor 5	
Factor 1						0.89
Tiredness	0.87					
Lack of vitality	0.82					
Lack of energy	0.81					
Muscle weakness	0.78					
Dry mouth/thirst	0.73					
Vertigo	0.63					
Headache	0.62					
Factor 2						0.85
Chest pain		0.84				
Shortness of breath		0.83				
Dyspnea		0.77				
Chest tightness		0.75				
Arrhythmia		0.65				
Lack of appetite		0.62				
Factor 3						0.80
Waking in night			0.82			
Trouble falling asleep			0.78			
Itchy skin			0.75			
Factor 4						0.77
Joint pain				0.88		
Sore muscles				0.80		
Numbness				0.78		
Factor 5						0.73
Vomiting					0.87	
Nausea					0.80	
Cramps					0.66	
Total scale						0.93

Factor 1, insufficient energy/vitality, had seven items, with factor loading ranging from 0.62 to 0.87, accounting for 41.2% of the variance. This factor reflected the symptoms of tiredness, headaches, muscle weakness, lack of energy and vertigo.

Factor 2, cardiac–pulmonary symptoms, had six items, with factor loading ranging from 0.62 to 0.84, accounting for 7.3% of the variance. This factor reflected the cardiopulmonary symptoms of chest pain, shortness of breath, dyspnea, and chest tightness.

Factor 3, sleep disturbances, contained three items, with factor loading ranging from 0.75 to 0.82, accounting for 6.7% of the variance. This factor reflected the trouble falling asleep or waking in the night.

Factor 4, musculoskeletal symptoms, had three items, with factor loading ranging from 0.78 to 0.88, accounting for 5.6% of the variance. This factor reflected problems such as muscle numbness and joint pain.

Factor 5, gastrointestinal distress, had three items, with factor loading ranging from 0.66 to 0.87, accounting for 4.7% of the variance. This factor reflected the gastrointestinal symptoms of vomiting and nausea.

### Reliability

After factor structure was confirmed, the investigators used Cronbach’s alpha coefficient to assess the reliability of the total scale and the factor-based subscales. Cronbach’s alpha coefficient for the final version of the HSD-22 total scale was 0.93, and the subscale alpha coefficients ranged from 0.73 to 0.89. In this study, twenty participants were selected who were over 20 years old had been undergoing HD for ≥3 months and were able to comprehend and communicate in Mandarin Chinese or Taiwanese to retest the stability of the HSD-22 questionnaire by measuring the test-retest reliability in the third weeks. The intraclass correlation coefficient (ICC) was used for the test-retest reliability, it was 0.92 (*p < 0.001*).

## Discussion

This study identified five factors of the Symptom Cluster via EFA. These five factors were: insufficient energy/vitality, cardiac–pulmonary distress, sleep disturbances, musculoskeletal distress, and gastrointestinal distress which were similar to the clusters identified by Yu et al. [12] which included energy and sensory discomfort, gastrointestinal (GI) and cardiac–pulmonary symptoms, cardiovascular symptoms, and electrolyte imbalance. However, these four clusters identified by Yu et al. [12] had apparent problems with overlapping (cardiac– pulmonary symptoms, cardiovascular symptoms) and vague dimensions (electrolyte imbalance). Yu et al. used the Somatic Symptoms Disturbance Index (SSDI), and the Content Validity Index was 0.8 with Cronbach’s alpha of .86. This study used the Hemodialysis Symptom Distress Scale (HSD). The Content Validity Index was 0.89 with Cronbach’s alpha of 0.9. EFA factor loading of Yu et al. was between 0.50–0.81, which explained 63.54% of the total variance. Factor loading of this study was between 0.62–0.87, which explained 65.5% of the total variance. The reliability and validity of this study were better than the study of Yu et al. Furthermore, the characteristics of symptom distress verified in our study were much more similar to those dimensions of Gastrointestinal (GI), musculoskeletal, neurological, and sleep disturbance [7]. Comparing the symptoms clusters verified in our study with those identified by Yu et al. [12] and Chaiviboontham et al. [7], the primary difference was that our study separated sleep disturbances as a factor, due to sleep disturbances resulting from multiple influencing factors presented in patients receiving HD [23]. These influence factors may be related to certain symptoms of distress, such as Gastrointestinal (GI), musculoskeletal, neurological, and sleep disturbance [7], or sensory discomfort [12]. It may explain why sleep disturbance was not an independent dimension/cluster [12, 17].

Factor 1, ‘insufficient energy/vitality’ was one of the most troublesome distresses among the multiple symptoms experienced by patients receiving HD which was also found in previous studies [12, 24]. Insufficient energy/vitality may be related to renal anemia due to lack of erythropoietin and latent gastrointestinal bleeding [24, 25].

‘Cardiac–pulmonary symptoms’ presented in Factor 2 was a common symptom of distress, it often resulted from fluid overload.

The symptoms of Factor 3 included waking in the night, trouble falling asleep and itchy skin those clustered into a factor called ‘sleep disturbances’. Patients with ESKD often experienced restless leg syndrome which resulted from peripheral neuropathy, or deep itchiness which made patients feel that they had to keep moving their feet or walking to relieve the pain that resulted in interrupted sleep [26, 27].

Joint pain, sore muscles, and numbness were clustered to become a new dimension called ‘musculoskeletal symptoms’. Gastroparesis was a distress for patients receiving HD due to autonomic neuropathy; it prolonged the time to empty their stomach and caused discomfort symptoms such as nausea, vomiting, and lack of appetite [28]. Furthermore, uremic polyneuropathy may be another factor causing patients to cramp; the earliest symptom was muscle cramps in the lower limbs [26]. Therefore, vomiting, nausea, and cramps were synthesized into a factor called ‘gastrointestinal distress’.

Cronbach’s alpha coefficients for the HSD-22 total scale (0.93) and each of the five subscales (0.77–0.85) indicated that this newly-constructed instrument had a good internal consistency. The results of test–retest analysis showed that the HSD-22 was relatively stable over a 2–4 -week period.

Multiple symptoms usually occurred concurrently when patients receiving Hemodialysis. To provide an effective intervention for symptom distress, a physiologically robust measurement is needed to capture the essence of symptom clusters under a group of symptoms that may share common etiology or biomechanics. The HSD-22 developed in this study covered five factors via factor analysis. Among these five factors, each factor covered a cluster of symptoms which may share common etiology or biomechanics as discussed above. The HSD-22 was verified, and improved one of the symptom clusters identified by HPs before, therefore, it was a valid and reliable scale and can provide a useful clinical assessment tool for healthcare professionals (HPs) working in the HD unit to identify possible symptom clusters of patients undergoing HD. To achieve more efficacy in treatments, we suggest that clinical interventions should be considered in terms of the common mechanism of symptom clusters to release the symptoms of patients receiving HD.

### Limitations

However, participants in this study were recruited from a medical university hospital which possesses the largest hemodialysis center having approximately 940 HD patients who came from different cities of southern Taiwan. This study may not be possible to apply the instrument to all hemodialysis patients in Taiwan. Further studies need to recruit participants from throughout Taiwan through multiple medical hospitals to verify generality of the results. Moreover, we suggest conducting a confirmatory factor analysis (CFA) to further test its construct validity to confirm the factor structure /symptom clusters established in this study.

## Conclusions

In conclusion, Physiologic testing from this study indicated that the HSD-22 scale is valid and reliable. We suggest that this assessment tool can be employed to identify the symptom clusters of patients receiving HD in the clinical setting. Such identification enables HPs to efficiently provide interventions to release patients’ symptom distress.

## Supplementary Information

**Additional file 1.**

## Data Availability

The datasets used and/or analyzed for this study are available from the corresponding author on reasonable request.
