# Supplementary material for: Development and testing of the hemodialysis symptom distress scale (HSD-22) to identify the symptom cluster by using exploratory factor analysis
Source: BMC Nephrol. 2021 Apr 12;22:128. doi: 10.1186/s12882-021-02337-7 (PMC8042879; doi:10.1186/s12882-021-02337-7)
Supplement: Supplementary file 1 — Additional file 1. [file 12882_2021_2337_MOESM1_ESM.pdf]

Symptom Distress Inventory for Hemodialysis Patient (HSD)

This questionnaire is to understand the symptoms of hemodialysis patients. The following items are discomfort for hemodialysis patients due to uremia or hemodialysis. The types and degree of disturbances depends on each person. Please fill in the symptoms that you experienced in the past three months. The answers are divided into four categories:

1. Did this symptom occurred?
   □0 No (You can skip to the next question if you didn't have this symptom in the past three months.)
   □1 Yes (When did this symptom occurred often?____)
2. Occurrence frequency (How often did this symptom occurred?)
   □1 It rarely happened □2 It happened on occasion
   □3 It happened frequently □4 It happened almost everyday
3. Severity: Please choose from 1 to 10 points from the line below which represents the severity of the symptom you experienced. The larger number means the severity is harder.

mild symptoms 1 2 3 4 5 6 7 8 9 10 severe symptoms

4. Disturbance: Please choose from 1 to 10 points from the line below which represents the degree of disturbance of the symptom you experienced. The larger number means the disturbance is harder.

mild disturbance 1 2 3 4 5 6 7 8 9 10 extremely disturbance

1. Tiredness □0 Didn't happened (*Skip to the next question*)
□1 It has happened. (*When did this symptom occurred often?*________________________)
Occurrence frequency：□1 It rarely happened □2 It happened on occasion □3 It happened frequent
□4 It happened almost everyday
Severity:
mild symptoms 1 2 3 4 5 6 7 8 9 10 severe symptoms

Disturbance: (Please write down the impact of this symptom to your daily life______________________)
mild disturbance 1 2 3 4 5 6 7 8 9 10 extremely disturbance

2. **Dry mouth/thirst** □0 Didn't happened (*Skip to the next question*)
□1 It has happened. (*When did this symptom occurred often?*_________________)
Occurrence frequency：□1 It rarely happened □2 It happened on occasion □3 It happened frequent
□4 It happened almost everyday
Severity:
mild symptoms 1 2 3 4 5 6 7 8 9 10 severe symptoms

Disturbance: (*Please write down the impact of this symptom to your daily life*______________________)
mild disturbance 1 2 3 4 5 6 7 8 9 10 extremely disturbance

3. **Muscle weakness** ☐0 Didn't happened (*Skip to the next question*)

☐1 It has happened. (*When did this symptom occurred often?*_______________)

Occurrence frequency：☐1 It rarely happened ☐2 It happened on occasion ☐3 It happened frequent

☐4 It happened almost everyday

Severity:

mild symptoms 1 2 3 4 5 6 7 8 9 10 severe symptoms

Disturbance: (*Please write down the impact of this symptom to your daily life*______________________)

mild disturbance 1 2 3 4 5 6 7 8 9 10 extremely disturbance

4. **Lack of vitality** ☐0 Didn't happened (*Skip to the next question*)

☐1 It has happened. (*When did this symptom occurred often?*__________________)

Occurrence frequency：☐1 It rarely happened ☐2 It happened on occasion ☐3 It happened frequent

☐4 It happened almost everyday

Severity:

mild symptoms 1 2 3 4 5 6 7 8 9 10 severe symptoms

Disturbance: (*Please write down the impact of this symptom to your daily life*______________________)

mild disturbance 1 2 3 4 5 6 7 8 9 10 extremely disturbance

5. **Nausea** ☐0 Didn't happened (*Skip to the next question*)

☐1 It has happened. (*When did this symptom occurred often?*_________________________)

Occurrence frequency：☐1 It rarely happened ☐2 It happened on occasion ☐3 It happened frequent

☐4 It happened almost everyday

Severity:

mild symptoms 1 2 3 4 5 6 7 8 9 10 severe symptoms

Disturbance: (*Please write down the impact of this symptom to your daily life*______________________)

mild disturbance 1 2 3 4 5 6 7 8 9 10 extremely disturbance

6. **Headache** ☐0 Didn't happened (*Skip to the next question*)

☐1 It has happened. (*When did this symptom occurred often?*________________________)

Occurrence frequency：☐1 It rarely happened ☐2 It happened on occasion ☐3 It happened frequent

☐4 It happened almost everyday

Severity:

mild symptoms 1 2 3 4 5 6 7 8 9 10 severe symptoms

Disturbance: (*Please write down the impact of this symptom to your daily life*______________________)

mild disturbance 1 2 3 4 5 6 7 8 9 10 extremely disturbance

7. **Chest pain** ☐0 Didn't happened (*Skip to the next question*)

☐1 It has happened. (*When did this symptom occurred often?*______________________)

Occurrence frequency：☐1 It rarely happened ☐2 It happened on occasion ☐3 It happened frequent

☐4 It happened almost everyday

Severity:

mild symptoms 1 2 3 4 5 6 7 8 9 10 severe symptoms

Disturbance: (*Please write down the impact of this symptom to your daily life*______________________)

mild disturbance 1 2 3 4 5 6 7 8 9 10 extremely disturbance

8. **Vomiting** ☐0 Didn't happened (*Skip to the next question*)

☐1 It has happened. (*When did this symptom occurred often?*_______________________)

Occurrence frequency：☐1 It rarely happened ☐2 It happened on occasion ☐3 It happened frequent

☐4 It happened almost everyday

Severity:

mild symptoms 1 2 3 4 5 6 7 8 9 10 severe symptoms

Disturbance: (*Please write down the impact of this symptom to your daily life*______________________)

mild disturbance 1 2 3 4 5 6 7 8 9 10 extremely disturbance

9. **Dyspnea** ☐0 Didn't happened (*Skip to the next question*)

☐1 It has happened. (*When did this symptom occurred often?*________________________)

Occurrence frequency：☐1 It rarely happened ☐2 It happened on occasion ☐3 It happened frequent

☐4 It happened almost everyday

Severity:

mild symptoms 1 2 3 4 5 6 7 8 9 10 severe symptoms

Disturbance: (*Please write down the impact of this symptom to your daily life*______________________)

mild disturbance 1 2 3 4 5 6 7 8 9 10 extremely disturbance

10. **Vertigo** ☐0 Didn't happened (*Skip to the next question*)

☐1 It has happened. (*When did this symptom occurred often?*______________________)

Occurrence frequency：☐1 It rarely happened ☐2 It happened on occasion ☐3 It happened frequent

☐4 It happened almost everyday

Severity:

mild symptoms 1 2 3 4 5 6 7 8 9 10 severe symptoms

Disturbance: (*Please write down the impact of this symptom to your daily life*_____________________)

mild disturbance 1 2 3 4 5 6 7 8 9 10 extremely disturbance

11. **Joint pain** ☐0 Didn't happened (*Skip to the next question*)

☐1 It has happened. (*When did this symptom occurred often?*____________________)

Occurrence frequency：☐1 It rarely happened ☐2 It happened on occasion ☐3 It happened frequent

☐4 It happened almost everyday

Severity:

mild symptoms 1 2 3 4 5 6 7 8 9 10 severe symptoms

Disturbance: (*Please write down the impact of this symptom to your daily life*_____________________)

mild disturbance 1 2 3 4 5 6 7 8 9 10 extremely disturbance

12. **Restless or jumpy legs** ☐0 Didn't happened (*Skip to the next question*)

☐1 It has happened. (*When did this symptom occurred often?*___________)

Occurrence frequency：☐1 It rarely happened ☐2 It happened on occasion ☐3 It happened frequent

☐4 It happened almost everyday

Severity:

mild symptoms 1 2 3 4 5 6 7 8 9 10 severe symptoms

Disturbance: (*Please write down the impact of this symptom to your daily life*_____________________)

mild disturbance 1 2 3 4 5 6 7 8 9 10 extremely disturbance

13. **Shortness of breath** ☐0 Didn't happened (*Skip to the next question*)

☐1 It has happened. (*When did this symptom occurred often?*______________)

Occurrence frequency：☐1 It rarely happened ☐2 It happened on occasion ☐3 It happened frequent

☐4 It happened almost everyday

Severity:

mild symptoms 1 2 3 4 5 6 7 8 9 10 severe symptoms

Disturbance: (*Please write down the impact of this symptom to your daily life*____________________)

mild disturbance 1 2 3 4 5 6 7 8 9 10 extremely disturbance

14. **Numbness** ☐0 Didn't happened (*Skip to the next question*)

☐1 It has happened. (*When did this symptom occurred often?*____________________)

Occurrence frequency：☐1 It rarely happened ☐2 It happened on occasion ☐3 It happened frequent

☐4 It happened almost everyday

Severity:

mild symptoms 1 2 3 4 5 6 7 8 9 10 severe symptoms

Disturbance: (*Please write down the impact of this symptom to your daily life*_____________________)

mild disturbance 1 2 3 4 5 6 7 8 9 10 extremely disturbance

15. **Lack of appetite** ☐0 Didn't happened (*Skip to the next question*)

☐1 It has happened. (*When did this symptom occurred often?*_______________)

Occurrence frequency：☐1 It rarely happened ☐2 It happened on occasion ☐3 It happened frequent

☐4 It happened almost everyday

Severity:

mild symptoms 1 2 3 4 5 6 7 8 9 10 severe symptoms

Disturbance: (*Please write down the impact of this symptom to your daily life*_____________________)

mild disturbance 1 2 3 4 5 6 7 8 9 10 extremely disturbance

16. **Sore muscles** ☐0 Didn't happened (*Skip to the next question*)

☐1 It has happened. (*When did this symptom occurred often?*_________________)

Occurrence frequency：☐1 It rarely happened ☐2 It happened on occasion ☐3 It happened frequent

☐4 It happened almost everyday

Severity:

mild symptoms 1 2 3 4 5 6 7 8 9 10 severe symptoms

Disturbance: (*Please write down the impact of this symptom to your daily life*_____________________)

mild disturbance 1 2 3 4 5 6 7 8 9 10 extremely disturbance

17. **Trouble falling asleep** ☐0 Didn't happened (*Skip to the next question*)

☐1 It has happened. (*When did this symptom occurred often?*____________)

Occurrence frequency：☐1 It rarely happened ☐2 It happened on occasion ☐3 It happened frequent

☐4 It happened almost everyday

Severity:

mild symptoms 1 2 3 4 5 6 7 8 9 10 severe symptoms

Disturbance: (*Please write down the impact of this symptom to your daily life*_____________________)

mild disturbance 1 2 3 4 5 6 7 8 9 10 extremely disturbance

18. **Itchy skin** ☐0 Didn't happened (*Skip to the next question*)
☐1 It has happened. (*When did this symptom occurred often?*______________________)
Occurrence frequency：☐1 It rarely happened ☐2 It happened on occasion ☐3 It happened frequent
☐4 It happened almost everyday

Severity:

mild symptoms 1 2 3 4 5 6 7 8 9 10 severe symptoms

Disturbance: (*Please write down the impact of this symptom to your daily life*_____________________)

mild disturbance 1 2 3 4 5 6 7 8 9 10 extremely disturbance

19. **Cramps** ☐0 Didn't happened (*Skip to the next question*)
☐1 It has happened. (*When did this symptom occurred often?*_____________________)
Occurrence frequency：☐1 It rarely happened ☐2 It happened on occasion ☐3 It happened frequent
☐4 It happened almost everyday

Severity:

mild symptoms 1 2 3 4 5 6 7 8 9 10 severe symptoms

Disturbance: (*Please write down the impact of this symptom to your daily life*_____________________)

mild disturbance 1 2 3 4 5 6 7 8 9 10 extremely disturbance

20. **Arrhythmia** ☐0 Didn't happened (*Skip to the next question*)
☐1 It has happened. (*When did this symptom occurred often?*__________________)
Occurrence frequency：☐1 It rarely happened ☐2 It happened on occasion ☐3 It happened frequent
☐4 It happened almost everyday

Severity:

mild symptoms 1 2 3 4 5 6 7 8 9 10 severe symptoms

Disturbance: (*Please write down the impact of this symptom to your daily life*_____________________)

mild disturbance 1 2 3 4 5 6 7 8 9 10 extremely disturbance

21. **Abdominal pain** ☐0 Didn't happened (*Skip to the next question*)
☐1 It has happened. (*When did this symptom occurred often?*________________)
Occurrence frequency：☐1 It rarely happened ☐2 It happened on occasion ☐3 It happened frequent
☐4 It happened almost everyday

Severity:

mild symptoms 1 2 3 4 5 6 7 8 9 10 severe symptoms

Disturbance: (*Please write down the impact of this symptom to your daily life*_____________________)

mild disturbance 1 2 3 4 5 6 7 8 9 10 extremely disturbance

22. **Lack of energy** ☐0 Didn't happened (*Skip to the next question*)
☐1 It has happened. (*When did this symptom occurred often?*________________)
Occurrence frequency：☐1 It rarely happened ☐2 It happened on occasion ☐3 It happened frequent
☐4 It happened almost everyday
Severity:
mild symptoms 1 2 3 4 5 6 7 8 9 10 severe symptoms

Disturbance: (*Please write down the impact of this symptom to your daily life*_____________________)
mild disturbance 1 2 3 4 5 6 7 8 9 10 extremely disturbance

23. **Constipation** ☐0 Didn't happened (*Skip to the next question*)
☐1 It has happened. (*When did this symptom occurred often?*__________________)
Occurrence frequency：☐1 It rarely happened ☐2 It happened on occasion ☐3 It happened frequent
☐4 It happened almost everyday
Severity:
mild symptoms 1 2 3 4 5 6 7 8 9 10 severe symptoms

Disturbance: (*Please write down the impact of this symptom to your daily life*_____________________)
mild disturbance 1 2 3 4 5 6 7 8 9 10 extremely disturbance

24. **Lack of sexual interest** ☐0 Didn't happened (*Skip to the next question*)
☐1 It has happened. (*When did this symptom occurred often?*_____________)
Occurrence frequency：☐1 It rarely happened ☐2 It happened on occasion ☐3 It happened frequent
☐4 It happened almost everyday
Severity:
mild symptoms 1 2 3 4 5 6 7 8 9 10 severe symptoms

Disturbance: (*Please write down the impact of this symptom to your daily life*_____________________)
mild disturbance 1 2 3 4 5 6 7 8 9 10 extremely disturbance

25. **Chest tightness** ☐0 Didn't happened (*Skip to the next question*)
☐1 It has happened. (*When did this symptom occurred often?*________________)
Occurrence frequency：☐1 It rarely happened ☐2 It happened on occasion ☐3 It happened frequent
☐4 It happened almost everyday
Severity:
mild symptoms 1 2 3 4 5 6 7 8 9 10 severe symptoms

Disturbance: (*Please write down the impact of this symptom to your daily life*_____________________)
mild disturbance 1 2 3 4 5 6 7 8 9 10 extremely disturbance

26. **Waking in night** ☐0 Didn't happened (*Skip to the next question*)
☐1 It has happened. (*When did this symptom occurred often?*________________)

Occurrence frequency：☐1 It rarely happened ☐2 It happened on occasion ☐3 It happened frequent
☐4 It happened almost everyday

Severity:

mild symptoms 1 2 3 4 5 6 7 8 9 10 severe symptoms

Disturbance: (*Please write down the impact of this symptom to your daily life*_____________________)

mild disturbance 1 2 3 4 5 6 7 8 9 10 extremely disturbance

Please write the symptom that you had which are not listed above in the space below.

Other symptom:

1. ____
2. ____
3. ____
4. ____
5. ____
